# The association between subjective–objective discrepancies in sleep duration and mortality in older men

**DOI:** 10.1038/s41598-022-22065-8

**Published:** 2022-11-04

**Authors:** Tomohiro Utsumi, Takuya Yoshiike, Yoshitaka Kaneita, Sayaka Aritake-Okada, Kentaro Matsui, Kentaro Nagao, Kaori Saitoh, Rei Otsuki, Masahiro Shigeta, Masahiro Suzuki, Kenichi Kuriyama

**Affiliations:** 1 Department of Sleep-Wake Disorders, National Institute of Mental Health, National Center of Neurology and Psychiatry, 4-1-1 Ogawa-Higashi, Kodaira, Tokyo, 187-8553 Japan; 2 Department of Psychiatry, The Jikei University School of Medicine, Tokyo, Japan; 3 Division of Public Health, Department of Social Medicine, Nihon University School of Medicine, Tokyo, Japan; 4 Department of Health Sciences, Saitama Prefectural University, Saitama, Japan; 5 Department of Clinical Laboratory, National Center Hospital, National Center of Neurology and Psychiatry, Tokyo, Japan; 6 Department of Psychiatry, National Center Hospital, National Center of Neurology and Psychiatry, Tokyo, Japan; 7 Department of Psychiatry, Nihon University School of Medicine, Tokyo, Japan

**Keywords:** Sleep, Perception, Public health, Geriatrics, Predictive markers

## Abstract

A discrepancy in subjective and objective estimations of sleep duration, which often diverge, could have long-term adverse effects on health outcomes in older adults. Using data from 2674 older adult men (≥ 65 years of age) of the Osteoporotic Fractures in Men Sleep Study, we assessed the longitudinal association between misperception index (MI), calculated as MI = (objective sleep duration – subjective sleep duration)/objective sleep duration, and all-cause mortality. During the follow-up with a mean (standard deviation) of 10.8 (4.2) years, 1596 deaths were observed. As a continuous variable, MI showed a linear relationship with all-cause mortality after adjusting for multiple covariates, including polysomnography-measured objective sleep duration [fully adjusted hazard ratio (HR), 0.69; 95% confidence interval [CI], 0.56–0.84]. As a categorical variable, the lowest MI quartile (vs. the interquartile MI range) was associated with increased mortality (fully adjusted HR, 1.28; 95% CI, 1.12–1.46), whereas the highest MI quartile was not associated with mortality (fully adjusted HR, 0.97; 95% CI, 0.85–1.11). The subjective overestimation of sleep duration may be a risk factor for all-cause mortality in older men. Future studies should examine why subjective overestimation of sleep duration is associated with all-cause mortality from a physiological perspective.

## Introduction

Older adults have higher prevalence rates of various sleep disorders, including insomnia^1^, sleep-related breathing^2,3^, and sleep-related movement disorders^4^, than other age groups. In addition, inadequate sleep in older adults is associated with an increased risk of developing physical ailments^5–7^, psychiatric disorders^8–10^, and all-cause mortality^9–11^. Therefore, it is a matter of public health to determine ways of optimizing their sleep schedules. While numerous epidemiological studies have relied on subjective measures of sleep duration (e.g., sleep logs or sleep-assessment questionnaires) to investigate various associations between sleep and health outcomes^12–18^, there are clear advantages to objective measures, which more accurately reflect the risk of physiological sleep loss. For example, studies using polysomnography (PSG), or actigraphy, have found evidence of frequent mismatches between subjective and objective sleep duration, especially in older adults^19,20^. Known as subjective–objective discrepancies in sleep duration, these mismatches may have important connotations for sleep physiology.

Such discrepancies are thought to fall along a single dimension ranging from subjective underestimation (subjective sleep duration < objective sleep duration) to subjective overestimation (subjective sleep duration > objective sleep duration)^21^; both extremes may impact longitudinal health outcomes. For example, subjective underestimation is considered a hallmark of insomnia, the most prominent phenotype of which is paradoxical insomnia, wherein extremely diminished subjective sleep duration is observed without any particular shortening of objective sleep duration^22–27^. Insomnia is of particular concern due to its associations with mental, metabolic, and cardiovascular diseases^28,29^. Similar physiological characteristics (e.g., cortical activity patterns during rapid eye movement sleep) that could be associated with adverse health outcomes in older adults^30^ have been reported in individuals with insomnia^31^ and underestimators of sleep duration in a general population^16,32^. However, while individuals with insomnia show a significant tendency to underestimate their sleep duration, they do not uniformly do so, and a significant portion of them rather overestimate than underestimate their sleep duration^18,33,34^, and the distribution of subjective–objective discrepancy resembles that of a general population^35^. This suggests that underestimation of sleep duration constitutes a distinct entity that involves both those with and without insomnia at large^32^ and could deteriorate health conditions through different physiological mechanisms in the general population. Thus, it is reasonable to assume that the subjective underestimation of sleep duration is associated with various adverse health outcomes in older adults.

At the other end of the spectrum, there is a lack of epidemiological and clinical knowledge on the subjective overestimation of sleep duration, although evidence suggests it may be associated with psychophysiological factors that are linked to poor health outcomes. Studies have shown that sleep overestimators have an increased propensity for both daytime sleep^21,36^ and decreased rapid eye movement (REM) activity^32^. In other research, individuals who reported sufficient amounts of sleep despite sleeping for short durations experienced less REM time than individuals who reported sufficient sleep amounts and objectively slept for long durations^37,38^. In sum, the literature suggests that overestimators tend to experience less REM sleep. In one case, an overestimator who reported sufficient subjective sleep continued to complain of daytime sleepiness and tiredness for a period lasting more than three years; contrary to the patient’s assumptions, objective measurements revealed short sleep durations and long periods of mid-sleep awakening^36^, both of which are associated with poor health outcomes^14,17,18,39^. Moreover, recent investigations have shown that low or “decreased percentage of REM sleep” is associated with a higher risk of all-cause mortality^30,40^. These findings suggest that subjective overestimations of sleep duration affect longitudinal health outcomes in relation to adverse changes in objective sleep variables that may become more evident in older adults (e.g., reduced sleep continuity and/or diminished REM sleep)^41^. Although there is scant evidence on gender differences in subjective–objective discrepancies in sleep duration, the subjective overestimation of sleep duration may be more directly influenced by physiological factors in men when compared to women. In a previous study, older men had better perception of their subjective sleep states when compared to older women, but had objectively worse sleep recordings, as evidenced by decreases in PSG-measured total sleep time, sleep efficiency, and the percentage of REM sleep^42–44^. Moreover, overestimation in men has been reported to be less closely associated with psychosocial factors than in women^45^. For older men, these findings indicate that the tendency to subjectively overestimate sleep despite possible objective deterioration is more directly linked to physiological factors than psychosocial factors. We therefore hypothesize that the subjective overestimation of sleep duration represents poorer current physiological conditions in older men, thus affecting their longitudinal health outcomes.

To test our hypothesis, we assessed longitudinal associations between subjective–objective discrepancies in sleep duration and all-cause mortality in a sample of older men, wherein data were obtained from the Osteoporotic Fractures in Men (MrOS) study^46,47^ and MrOS Sleep study^48,49^. In these cases, sleep was measured via both PSG (objective) and subjective perceptions. To assess the underestimation and overestimation of sleep duration, ratios (e.g. misperception index; MI)^32,50,51^ or differences (e.g. discrepancy index)^21,36,45,52^ between subjective and objective sleep duration have been utilized in previous studies. While the difference provides an absolute value of time duration and makes it easier to understand intuitively the degree of discrepancy, interindividual differences in sleep duration may influence the degree of discrepancy. To overcome this shortcoming of the discrepancy variables, we utilized the MI, calculated as [objective total sleep time (TST) minus subjective TST] divided by objective TST, which has been globally used as an indicator of subjective and objective discrepancy in sleep duration^53,54^.

## Results

### Sample characteristics

**Figure 1:**
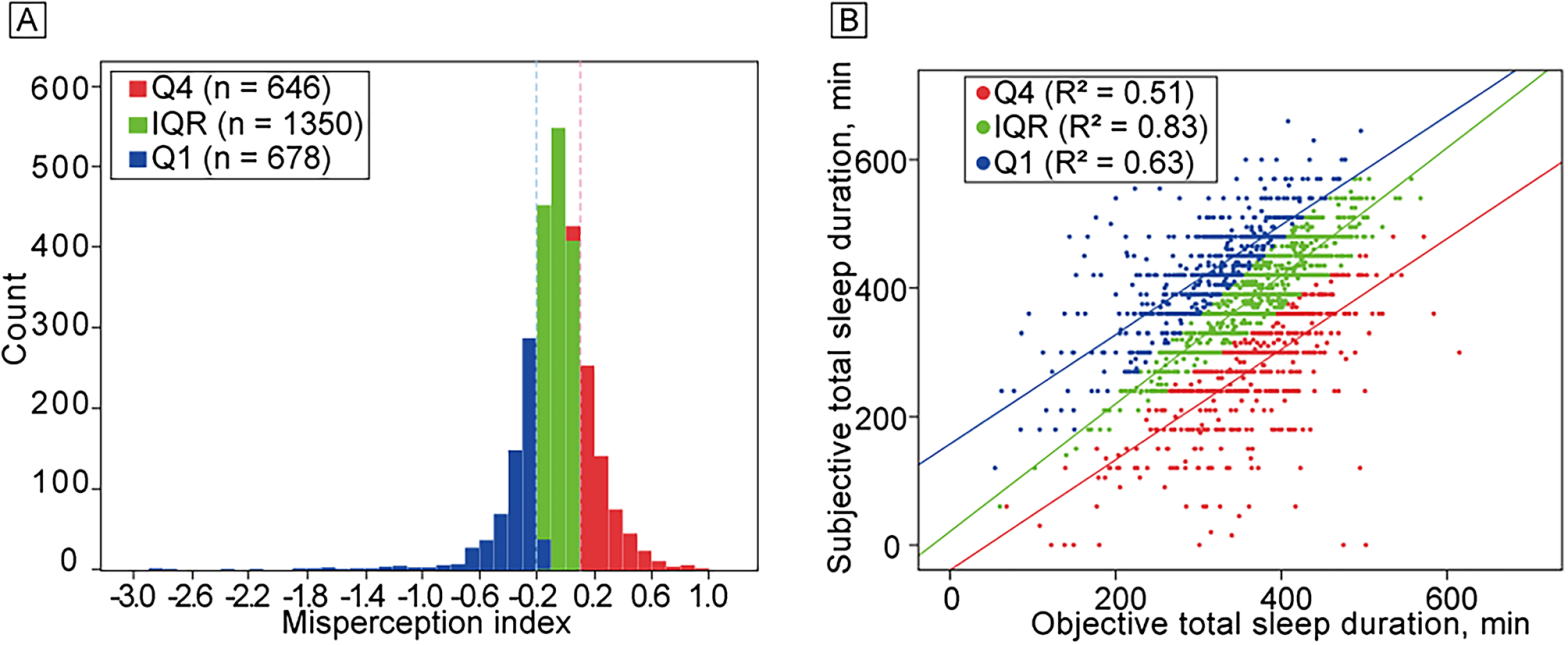
MI details for all participants**.** (**A**) MI distribution across the sample; (**B**) correlations between subjective total sleep duration and objective total sleep duration for each MI group. Low MI is shown in blue (overestimators: Q1, MI ≤ −0.19, n = 678), intermediate MI is shown in green (normoestimators: IQR, −0.19 to < 0.08, n = 1350), and high MI is shown in red (underestimators: Q4, MI > 0.08, n = 646). *IQR*  interquartile range, *Q1*  lowest quartile, *Q4*  highest quartile, *MI*  misperception index.

We analyzed a total of 2674 participants with a mean [standard deviation (SD)] age of 76.3 (5.5) years at baseline, followed-up for a mean (SD) of 10.8 (4.2) years. Mean (SD) objective sleep duration (objective total sleep time, oTST) and subjective sleep duration (subjective total sleep time, sTST) were 355.9 (69.0) and 371.9 (91.8) minutes, respectively, both normally distributed. Mean (SD) MI was − 0.07 (0.29), with normal distribution (Fig. 1A). Figure 1B shows correlations between sTST and oTST according to type.

Table 1 lists background, health status, and lifestyle information. The low MI group was older on average and had lower cognitive function and Physical Activity Scale for the Elderly (PASE) scores. Table 2 lists sleep characteristics. For objective assessments, the low MI group tended toward less REM sleep, shorter sleep times, longer sleep wake after sleep onset, shorter sleep efficiency, and more periodic limb movements (PLM); for subjective assessments, they had better sleep evaluations and longer sleep durations. Mean (SD) subjective–objective total sleep time difference was − 90.6 (60.3) in high MI group, 19.6 (27.4) in intermediate MI group, and 110.5 (46.2) in low MI group.

**Table 1:** Baseline demographic and health characteristics (complete case analysis).

Characteristic	No. (%)			
	High MI (> 0.08), n = 646	Intermediate MI (−0.19 to 0.08), n = 1350	Low MI (≤ −0.19), n = 678	All participants, n = 2674
Age, mean (SD), years	76.1 (5.5)	75.9 (5.3)	77.3 (5.6)	76.3 (5.5)
**Race**				
White	584 (90.4)	1242 (92.0)	616 (90.9)	2442 (91.3)
Other	62 (9.6)	108 (8.0)	62 (9.1)	232 (8.7)
**Education**				
≤ High school degree	165 (25.5)	261 (19.3)	141 (20.8)	567 (21.2)
≥ Some college	481 (74.5)	1089 (80.7)	537 (79.2)	2107 (78.8)
Body mass index	27.3 (3.8)	27.1 (3.7)	27.2 (4.0)	27.2 (3.8)
**Smoking status**				
Never	259 (40.1)	540 (40.0)	255 (37.7)	1054 (39.4)
Past	372 (57.6)	785 (58.1)	409 (60.4)	1566 (58.6)
Current	15 (2.3)	25 (1.9)	13 (1.9)	53 (2.0)
Daily caffeine intake, mean (SD), mg/dl	235.2 (243.1)	230.2 (240.4)	248.4 (262.4)	236.0 (246.8)
**Alcohol use**				
No alcohol (non-drinker)	234 (36.3)	459 (34.2)	221 (32.8)	914 (34.3)
Less than one drink per week	91 (14.1)	167 (12.4)	77 (11.4)	335 (12.6)
1–2 drinks per week	82 (12.7)	163 (12.1)	90 (13.4)	335 (12.6)
3–5 drinks per week	92 (14.3)	215 (16.0)	103 (15.3)	410 (15.4)
6–13 drinks per week	114 (17.7)	257 (19.1)	149 (22.1)	520 (19.5)
14 or more drinks per week	32 (5.0)	83 (6.2)	33 (4.9)	148 (5.6)
**Current medication use**				
Antidepressant	44 (6.8)	116 (8.6)	41 (6.0)	201 (7.5)
Benzodiazepine	31 (4.8)	61 (4.5)	31 (4.6)	123 (4.6)
Sleep medications	88 (13.6)	159 (11.8)	72 (10.6)	319 (11.9)
Hypertension	323 (50.0)	670 (49.6)	340 (50.2)	1333 (49.9)
Stroke	22 (3.4)	50 (3.7)	28 (4.1)	100 (3.7)
Heart attack	112 (17.3)	232 (17.2)	121 (17.9)	465 (17.4)
Transient ischemic attack	57 (8.8)	124 (9.2)	75 (11.1)	256 (9.6)
Congestive heart failure	47(7.3)	77 (5.7)	38 (5.6)	162 (6.1)
Type 2 diabetes	91 (14.1)	186 (13.8)	78 (11.5)	355 (13.3)
Chronic obstructive pulmonary disease	39 (6.0)	64 (4.7)	38 (5.6)	141 (5.3)
3 TMS score (0–100), mean (SD)	92.7 (6.6)	93.3 (5.5)	92.1(6.5)	92.9 (6.1)
Physical Activity Scale for the Elderly Score (0–400), mean (SD)	142.9 (74.0)	149.1 (70.8)	140.9(67.7)	145.5 (70.9)
Geriatric Depression Score (0–15), mean (SD)	2.0 (2.3)	1.6 (2.1)	1.6(2.0)	1.7 (2.1)
Follow-up time from sleep visit (year), mean (SD)	11.1 (4.1)	11.2 (4.1)	9.8(4.4)	10.8 (4.2)

*MI*  misperception index, *3TMS* Teng Mini-Mental State Examination.

Body mass index was calculated as weight in kilograms divided by height in meters squared.

**Table 2:** Baseline sleep characteristics (complete case analysis).

Characteristic	No. (%)			
	High MI (> 0.08), n = 646	Intermediate MI (−0.19 to 0.08), n = 1350	Low MI (≤ −0.19), n = 678	All participants, n = 2674
***Objective sleep measures, mean (SD)***				
**Stage N1**				
Sleep, % time	7.1 (4.7)	6.3 (3.8)	7.7 (4.7)	6.8 (4.3)
Sleep, min	25.2 (14.4)	22.7 (12.6)	24.1 (14.2)	23.7 (13.5)
**Stage N2**				
Sleep, % time	63.3 (9.2)	62.4 (9.5)	63.1 (10.3)	62.8 (9.6)
Sleep, min	232.6 (54.9)	229.9 (49.9)	200.4 (53.1)	223.1 (53.6)
**Stage N3**				
Sleep, % time	10.7 (8.5)	11.6 (9.0)	11.2 (9.4)	11.3 (9.0)
Sleep, min	40.2 (32.4)	43.0 (33.9)	35.5 (29.5)	40.4 (32.6)
**Stage REM**				
Sleep, % time	19.1 (6.9)	19.9 (6.3)	18.2 (6.8)	19.3 (6.6)
Sleep, min	71.4 (30.1)	74.3 (27.9)	58.9 (25.9)	69.7 (28.7)
Time in bed, min	503.2 (72.8)	482.9 (71.2)	470.6 (83.6)	484.7 (75.8)
Total sleep time, min	368.4 (71.2)	369.1 (60.6)	317.7 (68.5)	355.9 (69.0)
Wake after sleep onset, min	123.7 (71.7)	102.5 (61.3)	131.6 (67.3)	115.0 (66.7)
Sleep efficiency, %	75.4 (12.7)	78.9 (10.8)	71.4 (12.0)	76.2 (12.0)
REM latency, min	120.1 (86.2)	104.1 (71.5)	108.4 (80.1)	109.0 (77.7)
Overall arousal index	24.3 (12.4)	22.6 (11.3)	25.4 (12.1)	23.7 (11.8)
Apnea–hypopnea index at ≥ 3% desaturation	18.5 (16.3)	16.3 (14.3)	17.3 (15.8)	17.1 (15.2)
Periodic limb movements index	33.5 (36.9)	33.9 (35.8)	42.3 (42.1)	36.0 (37.9)
Periodic limb movements with arousals index	4.0 (5.6)	3.7 (5.4)	5.0 (6.7)	4.1 (5.8)
Sleep time with saturated oxygen below 80%, %time	0.09 (0.55)	0.09 (0.52)	0.09 (0.47)	0.09 (0.52)
**Self-reported sleep measures, mean (SD)**				
ESS score (0–24)	6.1 (3.8)	6.3 (3.6)	5.9 (3.8)	6.2 (3.7)
Pittsburgh Sleep Quality Index total score	6.9 (3.6)	5.4 (3.1)	4.7 (2.9)	5.6 (3.3)
Habitual bedtime	22:44:46 (1:09:13)	22:41:35 (1:06:03)	22:32:02 (1:12:49)	22:39:56 (1:08:44)
Habitual waketime	6:45:28 (1:17:28)	6:46:19 (1:10:33)	6:56:18 (1:15:41)	6:48:39 (1:13:42)
Total sleep time, at PSG, min	277.9 (85.3)	388.6 (66.3)	428.1 (73.7)	371.9 (91.8)
Subjective–objective total sleep time, difference, mean (SD), min	−90.6 (60.3)	19.6 (27.4)	110.5 (46.2)	16.0 (82.5)
Misperception index, mean (SD)	0.25 (0.17)	−0.05 (0.07)	−0.39 (0.31)	−0.07 (0.29)

*MI*  misperception index, *REM*  rapid eye movement, *ESS*  Epworth Sleepiness Scale, *PSG*  polysomnography.

### Associations between misperception index and all-cause mortality

**Figure 2:**
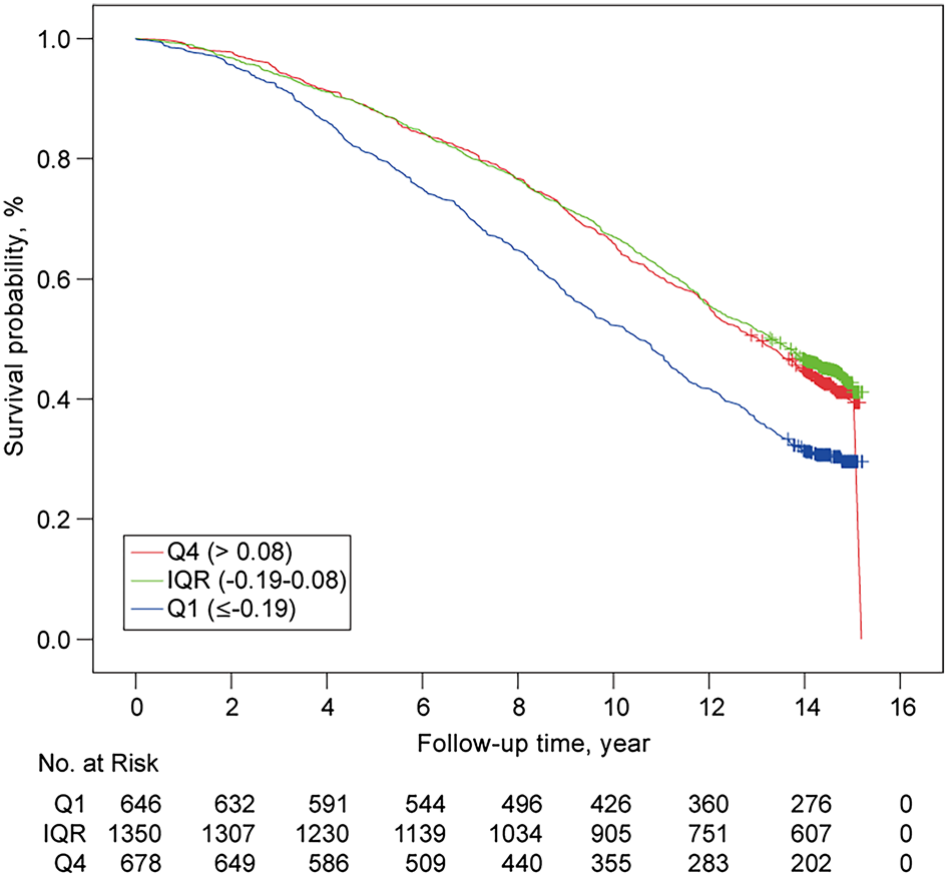
Unadjusted Kaplan–Meier Plots by MI. Survival probabilities from unadjusted Kaplan–Meier plots. *IQR* interquartile range, *Q1*  lowest quartile, *Q4*  highest quartile, *MI*  misperception index.

A total of 1596 deaths (59.7%) were reported over a mean (SD) follow-up of 10.8 (4.2) years. The regression analyses with MI as a continuous variable consistently showed a linear relationship between MI and all-cause mortality; as MI increased, all-cause mortality decreased [fully adjusted hazard ratio (HR), 0.69; 95% confidence interval (CI), 0.56–0.84]. The regression analyses with MI as a categorical variable showed that low MI (overestimation) was consistently associated with higher mortality when compared to intermediate MI (fully adjusted HR, 1.28; 95% CI, 1.12–1.46), while no such association was found between low MI (underestimation) and mortality (fully adjusted HR, 0.97; 95%CI, 0.85–1.11) (Table 3; Fig. 2).

**Table 3:** Mortality HRs from Cox regression by MI.

Predictor	Overall deaths, no. (%)	HR (95% CI)			
		Unadjusted	Age adjusted	Model 1^a^	Model 2^b^
MI as continuous variable	1596 (59.7)	0.57 (0.48–0.67)	0.65 (0.55–0.77)	0.67 (0.57–0.79)	0.69 (0.56–0.84)
**MI as categorical variable**					
Q4 (> 0.08)	374 (57.9)	1.05 (0.93–1.19)	1.04 (0.92–1.17)	1.01 (0.90–1.15)	0.97 (0.85–1.11)
IQR (−0.19 to 0.08)	750 (55.6)	Reference	Reference	Reference	Reference
Q1 (≤ −0.19)	472 (69.6)	1.51 (1.35–1.70)	1.37 (1.22–1.54)	1.38 (1.23–1.55)	1.28 (1.12–1.46)

*MI* misperception index.

^a^Model 1 included age, race (white or non-white), education, body mass index, smoking status, weekly alcohol consumption, daily caffeine use, antidepressants, benzodiazepines, sleep medications, and site.

^b^Model 2 included Model 1 plus overall arousal index, percentage of sleep time oxygen saturation below 80%, Stage 2%, Stage 3–4%, Stage REM%, Epworth Sleepiness Scale score, Pittsburgh Sleep Quality Index, Teng Mini-Mental State Examination score, Physical Activity Scale for the Elderly score, Geriatric Depression score, congestive heart failure, chronic obstructive pulmonary disease, diabetes, heart attack, stroke, wake after sleep onset, objective total sleep time, hypertension, apnea hypopnea index, periodic limb movements index, habitual bed time, habitual wake-up time, and subjective sleep wellness for the PSG night.

### Sensitivity analysis

We conducted several sensitivity analyses to exclude other possible explanations for the association between MI and all-cause mortality. The sensitivity analyses with MI as a continuous variable consistently showed a linear relationship between increased MI and decreased all-cause mortality; that is, excluding death within two years (fully adjusted HR, 0.72; 95% CI, 0.58–0.89) (Table S1), apnea–hypopnea index scores greater than 30 (fully adjusted HR, 0.68; 95% CI, 0.53–0.85) (Table S2), sleeping pills, benzodiazepines, and/or antidepressants (fully adjusted HR, 0.68; 95% CI, 0.54–0.87) (Table S3), depression (fully adjusted HR, 0.74; 95% CI, 0.59–0.93) (Table S4), objectively short/long sleep durations (fully adjusted HR, 0.53; 95% CI, 0.35–0.82) (Table S5), PSG sleep ratings of extremely poor or better than normal (fully adjusted HR, 0.65; 95% CI, 0.50–0.85) (Table S6), and subjective sleep complaints (fully adjusted HR, 0.60; 95% CI, 0.42–0.85) (Table S7). Across the sensitivity analyses with MI as a categorical variable, low MI was consistently associated with higher mortality when compared to intermediate MI, while no such association was found between high MI and mortality (Tables S1-S7). With MI divided into quintiles, including Q’1 (≤ −0.23), Q’2 (−0.23 to ≤ −0.11), Q’3 (−0.11 to ≤ −0.01), Q’4 (−0.01 to ≤ 0.12), and Q’5 (> 0.12), the lowest quintile was significantly associated with higher mortality when compared to the intermediate quintile (Q’3) (fully adjusted HR, 1.29; 95% CI, 1.09–1.53) (Table S8).

Finally, the regression analyses with data supplemented via multiple imputation for missing values were consistent with MI, both in continuous and categorical form (Table S9).

## Discussion

We found a negative association between MI and all-cause mortality in a longitudinal cohort of older men. After dividing participants into subgroups by quartiles/quintiles, those with low (vs. intermediate) MI (overestimation) showed consistent associations with increased mortality, while those with high MI (underestimation) did not, thus indicating an increased mortality risk among sleep overestimators. The observed associations between MI and mortality persisted through a series of sensitivity analyses, suggesting that MI influenced mortality independent of the end-of-life functional change, sleep apnea, sleeping pills, depression, objective long/short sleep duration, extreme dissatisfaction/satisfaction with sleep during PSG, and/or subjective sleep complaints. In sum, low MI (the overestimation of sleep duration) may indicate a higher risk of mortality in older men.

Following previous studies, MI was normally distributed in this sample, with the extremes representing the subjective underestimation and overestimation of sleep duration. Subjective–objective sleep discrepancies have primarily been investigated through studies on subjective underestimations of sleep duration in insomniacs, which therein constitutes a behavioral hallmark known as sleep state misperception^22^. For example, Mercer et al. found that individuals with insomnia underestimated total sleep while overestimating wake compared to good sleepers^55^. Similarly, Manconi et al. found that insomniacs tended to underestimate sleep times while healthy counterparts gave accurate estimates^51^. On the other hand, Attarian et al. reported on overestimated sleep durations in older adult women, conceptualizing this as “reverse” sleep state misperception, thus providing important clues for future research into subjective–objective discrepancies^36^. Trajanovic et al. proposed that such discrepancies spanned a continuum ranging from underestimation to overestimation^21^. Here, the subjective overestimation of sleep duration may represent a distinct condition that can appear in healthy individuals and insomniacs^25,35,50,51,56^. These findings support the idea that MI reaches both ends of the continuum, thus suggesting the clinical and epidemiological importance of subjective overestimations.

Low MI (the overestimation of sleep duration) was significantly associated with increased all-cause mortality, independent of objective sleep variables that were also associated with increased mortality. Several mechanisms are assumed to exist for this relationship. While physiological sleep need may decrease with age, there is no clear reduction in time in bed^57^, suggesting excessive time in bed relative to physiological sleep need in older adults. Epidemiological evidence suggests that objective longer time in bed is associated with higher total mortality in older adults independent of objective sleep duration per se^17,58^. In addition, there is evidence linking objective long time in bed and self-reported long sleep duration^59^. Thus, overestimation could be associated with mortality through such discrepancy between physiological and behavioral (hygienic) sleep durations.

Moreover, overestimators may have several objectively poor sleep states, which may lead to increased mortality particularly via cardiovascular disease. First, it has been suggested that in the general population, overestimation of sleep duration is associated with decreased REM activity^32^, increased severity of obstructive sleep apnea^60^, and decreased sleep efficiency^34^. Meanwhile, in patients with sleep disorders, it has been suggested that the mean sleep latency in the multiple sleep latency test was significantly shorter in overestimators^21^ and the frequency of PLM was higher in the overestimators with insomnia^50^. Consistent with a range of previous reports, the low MI group showed comparatively lower rates of REM sleep, shorter sleep durations, lower sleep efficiency, and more PLM in the present sample. Furthermore, these objective factors have been reported to be associated with all-cause mortality. Objectively shorter sleep duration^61^, lower rates of REM sleep^30^, and more PLM^62^ have been associated with cardiovascular mortality, and higher AHI^63^ has been associated with cardiovascular and cerebrovascular mortality. Although the present study does not allow clarification of the causal mechanisms of the observed association, it is speculated that these physiological changes (e.g., decreased REM rate and sleep efficiency) may occur in the background in overestimators, leading to increased total mortality. Further physiological studies are needed to elucidate the mechanisms that underly overestimation of sleep duration in the general population in more detail. Unveiling the underlying mechanisms may further allow the chance to test whether a certain intervention to optimize the perception of sleep duration reduces mortality in the epidemiological setting.

Contrary to our hypothesis, the subjective underestimation of sleep duration was not associated with an increased risk of mortality. It is possible that underestimation of sleep duration was not associated with an increased risk of all-cause mortality because the underestimators in this study did not fully reflect the underestimators in the previous study. First, we did not analyze any data from older women, who appear more likely to complain of subjective sleep disturbances even when their objective sleep states are better than those of older men^42–44^. Thus, older women with high MI may experience more subjective sleep disturbances, which is suggestive of insomnia. Therefore, our null findings on the relationship between high MI and mortality may have differed if older women were included. This negative finding may also be attributable to the nonclinical characteristics of sleep abnormalities in the current sample. Clearly, the high MI group did not wholly represent chronic insomnia, as its members did not necessarily have sleep difficulties. Moreover, the definition for subjectively underestimated sleep duration is not confined to the context of chronic insomnia. Indeed, a previous review reported that underestimation was associated with other health problems, including depression and irritable bowel syndrome^24^. However, there is scant evidence that paradoxical insomnia, which can be a form of chronic insomnia that can be characterized by extremely high MI^25^, is independently associated with poor long-term health outcomes. Meanwhile, in examining the relationship between underestimation and poor health outcomes, it is also necessary to examine the mechanisms that cause underestimation. As objective factors involved in high MI, NREM and REM activity have been shown to be higher in the extreme underestimation group in the general population^32^. However, in the present study, there was no clear trend toward lower REM% in the low subject-object ratio group compared to the intermediate estimation group. Other findings in the general population are scant, but in their findings on insomnia, Parrino et al. reported higher arousal index in paradoxical insomniacs with extreme underestimation compared to healthy controls^64^. Furthermore, sleep duration underestimation is also important for mental health status, and is thought to be common in depression^65^ and insomnia^35^. Fernandez-Mendoza et al. also suggested that the insomnia underestimation group showed higher scores in depression and anxiety scales^34^. In the present study, in the general population, the underestimator group had a higher arousal index and slightly higher scores on the Geriatric Depression Scale (GDS) and Pittsburgh Sleep Quality Index (PSQI) than the intermediate estimator group. This is consistent with previous studies and may be associated with underestimation. Future studies should investigate the association between high MI and health outcomes and background physiological mechanism, particularly in individuals with chronic insomnia disorder as well as those without insomnia in the general older adult population.

### Strengths and limitations

This study investigated a large prospective cohort using a combination of validated objective/subjective sleep variables and PSG. Moreover, our results remained robust after adjusting for multiple confounders and conducting sensitivity analyses. However, there were also some limitations. First, the majority of participants were older Caucasians with preserved activities of daily living registered from the community, which limits generalizability. There may also be residual confounding factors such as sex, especially considering that older men and women may have different sociological background factors that are associated with the overestimation of sleep duration^45^. Second, we cannot completely exclude the influence of the first-night effect on both the objective and subjective estimation of sleep duration^66–68^, although this does not always occur with single-night PSG^69^. Further, reports involving consecutive PSG nights suggest that objective sleep duration does not always change significantly across recordings^70^. We also controlled for the effects of subjective sleep wellness on the PSG night as a covariate in the survival group, thus finding consistent results; our findings do not appear problematized by the first-night effect. Although further investigations are needed in other samples to determine the long-term adverse effects of subjective and objective discrepancy in sleep duration on health outcomes, this cohort study found an association between the subjective overestimation of sleep duration and all-cause mortality, thus positioning MI as an important determinant of longitudinal health outcomes in a sample of older men. Moreover, these findings challenge the efficacy of approaches that rely on basic sleep duration as a measure for improving public health.

## Methods

### Participants

As a community-based longitudinal observational investigation, the MrOS study was conducted among 5,994 participants from six centers in the United States between 2000 and 2002. All participants were at least 65 years of age and could walk independently. The study details have been previously published^46,47^. Of those participants, 3135 engaged in the ancillary MrOS Sleep study between 2003 and 2005, which included a comprehensive sleep assessment with in-home PSG recordings^48,49^. However, 224 had missing PSG data (i.e., 179 with no PSG recordings and 45 who failed to record PSG), which resulted in 2911 valid observations. In this study, we only included participants with verified survival or known causes of death through August 2019, which reduced the sample to 2675 subjects. Finally, one participant did not have data on subjective sleep time from the morning survey, which ultimately reduced the analyzed sample to 2674 subjects (Fig. S1). All analyzed data (including vital data) are available on the Internet, from both the National Sleep Research Resource website (http://sleepdata.org. [November 20, 2019])^40^ and MrOS online (https://mrosdata.sfcc-cpmc.net. [April 2, 2020]). For the MrOS study, ethical approval was obtained from the relevant local institutional review boards and all participants provided informed consent. The current study was approved by the Ethics Committee of the National Center of Neurology and Psychiatry, Tokyo, Japan and conducted in line with the principles of the Declaration of Helsinki.

### Measures

#### Objective sleep duration

An unattended, portable in-home PSG (Safiro, Compumedics, Inc., Melbourne, Australia) was recorded at baseline, with scoring based on the American Academy of Sleep Medicine rules^71^. Standard PSG characteristics were also assessed^48,49^. We defined PSG-measured total sleep time as oTST.

#### Subjective sleep duration

Following the night of their in-home PSG recordings, participants completed a morning survey, which asked “How much time do you think you actually slept last night?” to rate their subjective sleep duration in terms of the number of perceived hours and minutes achieved (sTST). Subjective responses to PSG recordings were evaluated via morning surveys in which participants evaluated their preceding night’s sleep along a 5-point scale ranging from “poor = 1” to “good = 5” (compared to habitual sleep).

#### Primary exposure

The primary exposures were the overestimation and underestimation of sleep duration, as compared to the intermediate estimation of sleep duration. As a measure of subjective–objective discrepancy in sleep duration, we used the misperception index, calculated as MI = (objective sleep duration – subjective sleep duration)/objective sleep duration, as continuous variable. In addition, we divided MI into three groups using quartiles, therein defining the lowest (Q1 ≤ −0.19) and highest MI quartiles (Q4 > 0.08) as high MI (underestimation) and low MI (overestimation), respectively; the interquartile range of MI (IQR; Q2–Q3, −0.19 to ≤ 0.08) served as the reference category (normal estimation). Participants with high MI (Q4 > 0.08) and low MI (Q1 ≤ −0.19) were considered underestimators and overestimators, respectively, while those with intermediate MI (IQR) were considered normoestimators.

#### Mortality outcome

Participants or their next of kin were contacted every four months after baseline to confirm survival^30^. Reported deaths were validated via death certificates^46,47^.

#### Other covariates

Participants reported a variety of baseline characteristics through self-administered questionnaires, interviews, and clinical examinations. Specific items included education, race/ethnicity, body mass index (BMI), smoking history, daily caffeine intake, and alcohol consumption. Medication use and medical history were assessed via questionnaire; medications included antidepressants, sleeping pills, and benzodiazepines that may affect sleep/sleep structure, while medical history included clinically diagnosed hypertension, stroke, myocardial infarction, transient ischemic attack, heart failure, type 2 diabetes, and chronic obstructive pulmonary disease (COPD). In addition, participants completed the 15-item GDS^72^, Modified Mini-Mental State Examination^73^, and PASE^74^. Self-administered sleep questionnaires included the Epworth Sleepiness Scale^75^, and PSQI^76^. Circadian sleep timing was evaluated based on PSQI-derived habitual bed times and wake-up times over the previous month^77–79^.

### Statistical analysis

Of the 2674 participants, 81 (3.03%) had missing values. We first conducted analyses in complete cases, then compared the results with those of the multiple assignment method. We used Cox proportional hazard models to evaluate the longitudinal associations between high MI (underestimation) or low MI (overestimation) and all-cause mortality. We first assessed MI as a continuous variable, since no categorical definitions were provided for the underestimation or overestimation of PSG-measured total sleep time. We then assessed MI as a categorical variable, therein defining the lowest (Q1 ≤ −0.19) and highest MI quartiles (Q4 > 0.08) as low MI (overestimation) and high MI (underestimation), respectively; the interquartile range of MI (IQR; Q2–Q3, −0.19 to ≤ 0.08) served as the reference category (normal estimation).

Cox models were run in the unadjusted model, age-adjusted model, and other two models (Model 1 and Model 2) using known covariates for adverse health outcomes. For Model 1, we used demographic covariates, including age, race/ethnicity, education, BMI, smoking status, alcohol, caffeine, medication use (sleeping pills, benzodiazepine, and/or antidepressants), and study sites. For Model 2, adding to Model 1 variables, we used health and sleep covariates, including circadian sleep timing (habitual bed times and wake-up times), Epworth Sleepiness Scale scores, PSQI score^33,34^, Teng Modified Mini-Mental State Examination scores, PASE scores, Geriatric Depression Scale scores, history of congestive heart failure, COPD, diabetes, hypertension, heart attack, and stroke, and PSG variables, including the overall arousal index, percentage of sleep time oxygen saturation below 80%, percentages of Stage N2, Stage REM%, wake after sleep onset, total sleep time, PLM index, and apnea–hypopnea index^30^; we also included the percentage of Stage N3, which may affect the perception of sleep duration^32,80^, and subjective sleep wellness on the PSG night^81^. The results were presented as HRs with 95% CIs. The proportional hazards assumptions were assessed using log minus log survival curve. All analyses were performed using SPSS Statistics, version 23 (IBM Japan, Tokyo), with significance set at a standard two-sided level of 5%.

### Sensitivity analysis

We conducted several analyses to exclude other possible explanations for the association between MI and all-cause mortality. First, participants with deaths within two years were excluded^82–84^. Second, participants with apnea–hypopnea index scores greater than 30 were excluded. Third, participants who used sleeping pills, benzodiazepines, and/or antidepressants were excluded. Fourth, depressed participants were excluded (i.e., Geriatric Depression Scale scores > 4 or antidepressant use). Fifth, the confounding effects of objectively short and long sleep durations were excluded, as represented by the lowest and highest quartiles, respectively (objective total sleep time < 317.0 min or ≥ 400.0 min from polysomnography). Sixth, participants who rated their sleep during PSG as extremely poor or far better than normal were excluded, as either rating may represent excessive responses to monitoring. Seventh, participants who complained of sleep disturbance (i.e., PSQI scores ≥ 6) were excluded so that the sample being examined was free from sleep complaints. To ensure that the association between MI and mortality did not solely depend on the definitions for high/low MI, we conducted another sensitivity analysis with MI as a categorical variable (quintiles).

Finally, we evaluated missing values. As such instances were considered random, we created 20 datasets and assigned missing values via the multiple assignment method, mainly using the chain method^85,86^. We then compared these results with those from the complete case analysis^86^.

## Supplementary Information

Supplementary Information.

## Data Availability

The data analyzed in this article are available through the National Sleep Research Resource website (http://sleepdata.org) and MrOS online (https://mrosonline.ucsf.edu).
