# Supplementary material for: The association between subjective–objective discrepancies in sleep duration and mortality in older men
Source: Sci Rep. 2022 Nov 4;12:18650. doi: 10.1038/s41598-022-22065-8 (PMC9636161; doi:10.1038/s41598-022-22065-8)
Supplement: Supplementary file 1 — Supplementary Information. [file 41598_2022_22065_MOESM1_ESM.pdf]

Supplementary Material

The association between subjective-objective discrepancies in sleep duration and mortality in older men

Tomohiro Utsumi, Takuya Yoshiike, Yoshitaka Kaneita, Sayaka Aritake-Okada, Kentaro Matsui, Kentaro Nagao, Kaori Saitoh, Rei Otsuki, Masahiro Shigeta, Masahiro Suzuki, Kenichi Kuriyama

Supplementary Figure and Table Captions

Figure S1 Flow Chart Illustrating Participant Selection From the Osteoporotic Fractures in Men Sleep Study Samples

Table S1 Mortality HRs From Cox Regression by MI for Participants who Survived the First Two Years of Study

Table S2 Mortality HRs From Cox Regression by MI for Participants Without Severe OSA

Table S3 Mortality HRs From Cox Regression by MI for Participants not Taking Medication

Table S4 Mortality HRs From Cox Regression by MI for Participants Without Depression

Table S5 Mortality HRs From Cox Regression by MI for Participants With Objective Intermediate Sleep Duration

Table S6 Mortality HRs From Cox Regression by MI for Participants Without Extremely Poor or Better Sleep Than Usual

Table S7 Mortality HRs From Cox Regression by MI for Participants Without Subjective Sleep Complaints

Table S8 Mortality HRs From Cox Regression by MI by Quintiles

Table S9 Mortality HRs From Cox Regression by MI After Multiple Imputation

Figure S1 Flow Chart Illustrating Participant Selection From the Osteoporotic Fractures in Men Sleep Study Samples

A total of 3,135 participants engaged in the MrOS Sleep study. Of these, 224 had missing PSG data, 236 had unknown causes of survival or death, and one had no subjective sleep time data from the morning survey, resulting in 2674 subjects in the final dataset.

Abbreviations: MrOS, the Osteoporotic Fractures in Men study; PSG, polysomnography

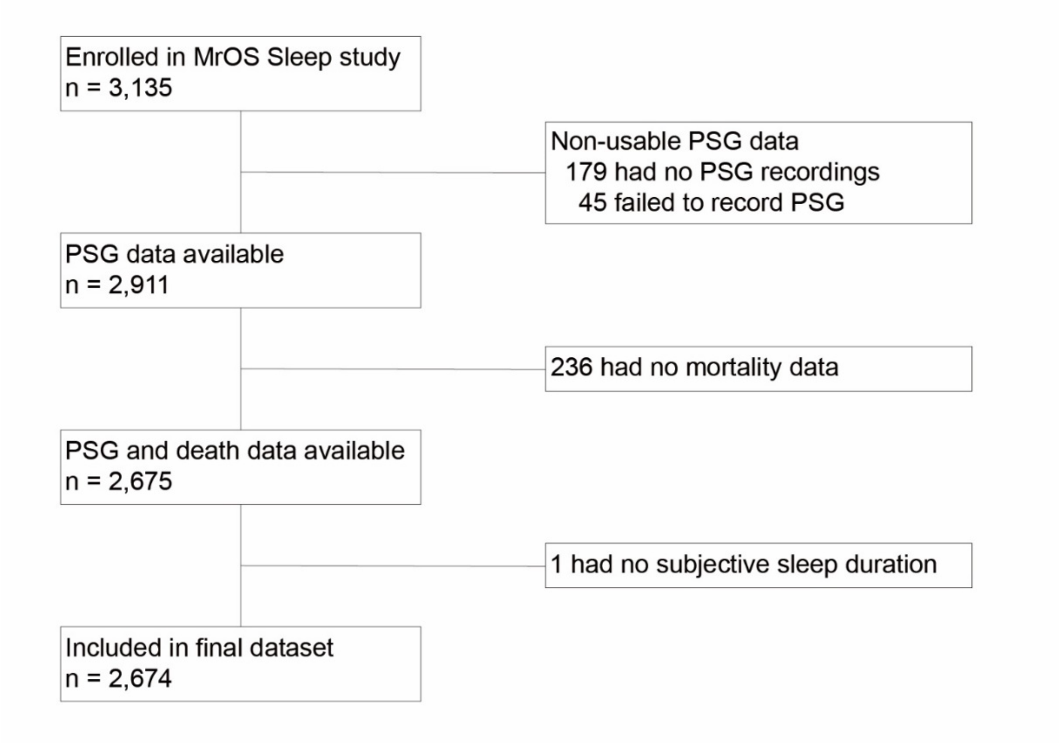

Table S1 Mortality HRs From Cox Regression by MI for Participants who Survived the First Two Years of Study

Predictor	Overall Deaths, No. (%)	HR (95% CI)			
		Unadjusted	Age adjusted	Model 1[a]	Model 2[b]
MI as continuous variable	1510 (58.3)	0.59 (0.49-0.70)	0.67 (0.56-0.79)	0.68 (0.58-0.81)	0.72 (0.58-0.89)
MI as categorical variable					
Q4 (> 0.08)	360 (57.0)	1.08 (0.95-1.22)	1.06 (0.94-1.21)	1.04 (0.92-1.18)	1.00 (0.87-1.14)
IQR (-0.19-0.08)	707 (54.1)	Reference	Reference	Reference	Reference
Q1 (≤-0.19)	443 (68.3)	1.52 (1.35-1.72)	1.39 (1.23-1.57)	1.40 (1.24-1.58)	1.29 (1.13-1.49)

[a] Model 1 included age, race (white or non-white), education, body mass index, smoking status, weekly alcohol consumption, daily caffeine use, antidepressants, benzodiazepines, sleep medications, and site.
[b] Model 2 included Model 1 plus overall arousal index, percentage of sleep time oxygen saturation below 80%, Stage 2%, Stage 3-4%, Stage REM%, Epworth Sleepiness Scale score, Pittsburgh Sleep Quality Index, Teng Mini-Mental State Examination score, Physical Activity Scale for the Elderly score, Geriatric Depression score, congestive heart failure, chronic obstructive pulmonary disease, diabetes, heart attack, stroke, wake after sleep onset, objective total sleep time, hypertension, apnea hypopnea index, periodic limb movements index, habitual bed time, habitual wake-up time, and subjective sleep wellness for the PSG night.
Abbreviation: MI = misperception index.

Table S2 Mortality HRs From Cox Regression by MI for Participants Without Severe OSA

Predictor	Overall Deaths, No. (%)	HR (95% CI) Unadjusted	Age adjusted	Model 1[a]	Model 2[b]
MI as continuous variable	1290 (58.1)	0.57 (0.48-0.69)	0.63 (0.52-0.75)	0.65 (0.54-0.78)	0.68 (0.53-0.85)
MI as categorical variable					
Q4 (> 0.08)	285 (55.2)	1.04 (0.90-1.19)	1.05 (0.91-1.20)	1.03 (0.89-1.18)	0.98 (0.85-1.14)
IQR (-0.19-0.08)	620 (54.4)	Reference	Reference	Reference	Reference
Q1 (≤-0.19)	385 (68.4)	1.52 (1.33-1.72)	1.44 (1.27-1.64)	1.43 (1.26-1.63)	1.30 (1.12-1.51)

[a] Model 1 included age, race (white or non-white), education, body mass index, smoking status, weekly alcohol consumption, daily caffeine use, antidepressants, benzodiazepines, sleep medications, and site.
[b] Model 2 included Model 1 plus overall arousal index, percentage of sleep time oxygen saturation below 80%, Stage 2%, Stage 3-4%, Stage REM%, Epworth Sleepiness Scale score, Pittsburgh Sleep Quality Index, Teng Mini-Mental State Examination score, Physical Activity Scale for the Elderly score, Geriatric Depression score, congestive heart failure, chronic obstructive pulmonary disease, diabetes, heart attack, stroke, wake after sleep onset, objective total sleep time, hypertension, apnea hypopnea index, periodic limb movements index, habitual bed time, habitual wake-up time, and subjective sleep wellness for the PSG night.
Abbreviation: MI = misperception index.

Table S3 Mortality HRs From Cox Regression by MI for Participants not Taking Medication

Predictor	Overall Deaths, No. (%)	HR (95% CI)			
		Unadjusted	Age adjusted	Model 1[a]	Model 2[b]
MI as continuous variable	1278 (58.1)	0.56 (0.46-0.68)	0.65 (0.54-0.79)	0.67 (0.56-0.81)	0.68 (0.54-0.87)
MI as categorical variable					
Q4 (> 0.08)	302 (57.4)	1.11 (0.96-1.27)	1.11 (0.97-1.28)	1.10 (0.96-1.26)	1.06 (0.91-1.23)
IQR (-0.19-0.08)	590 (53.3)	Reference	Reference	Reference	Reference
Q1 (≤-0.19)	386 (68.1)	1.54 (1.36-1.76)	1.44 (1.26-1.63)	1.44 (1.26-1.64)	1.33 (1.15-1.54)

[a] Model 1 included age, race (white or non-white), education, body mass index, smoking status, weekly alcohol consumption, daily caffeine use, and site.
[b] Model 2 included Model 1 plus overall arousal index, percentage of sleep time oxygen saturation below 80%, Stage 2%, Stage 3-4%, Stage REM%, Epworth Sleepiness Scale score, Pittsburgh Sleep Quality Index, Teng Mini-Mental State Examination score, Physical Activity Scale for the Elderly score, Geriatric Depression score, congestive heart failure, chronic obstructive pulmonary disease, diabetes, heart attack, stroke, wake after sleep onset, objective total sleep time, hypertension, apnea hypopnea index, periodic limb movements index, habitual bed time, habitual wake-up time, and subjective sleep wellness for the PSG night.
Abbreviation: MI = misperception index.
Medications included sleeping pills, benzodiazepines, and antidepressants.

Table S4 Mortality HRs From Cox Regression by MI for Participants Without Depression

Predictor	Overall Deaths, No. (%)	HR (95% CI)			
		Unadjusted	Age adjusted	Model 1[a]	Model 2[b]
MI as continuous variable	1314 (57.5)	0.56 (0.47-0.68)	0.67 (0.56-0.80)	0.68 (0.57-0.82)	0.74 (0.59-0.93)
MI as categorical variable					
Q4 (> 0.08)	295 (54.6)	1.03 (0.90-1.18)	1.04 (0.91-1.20)	1.02 (0.89-1.17)	0.99 (0.85-1.15)
IQR (-0.19-0.08)	612 (53.2)	Reference	Reference	Reference	Reference
Q1 (≤-0.19)	407 (68.4)	1.56 (1.38-1.77)	1.41 (1.24-1.60)	1.40 (1.23-1.59)	1.31 (1.14-1.51)

[a] Model 1 included age, race (white or non-white), education, body mass index, smoking status, weekly alcohol consumption, daily caffeine use, benzodiazepines, sleep medications, and site.
[b] Model 2 included Model 1 plus overall arousal index, percentage of sleep time oxygen saturation below 80%, Stage 2%, Stage 3-4%, Stage REM%, Epworth Sleepiness Scale score, Pittsburgh Sleep Quality Index, Teng Mini-Mental State Examination score, Physical Activity Scale for the Elderly score, Geriatric Depression score, congestive heart failure, chronic obstructive pulmonary disease, diabetes, heart attack, stroke, wake after sleep onset, objective total sleep time, hypertension, apnea hypopnea index, periodic limb movements index, habitual bed time, habitual wake-up time, and subjective sleep wellness for the PSG night.
Abbreviation: MI = misperception index.

Table S5 Mortality HRs From Cox Regression by MI for Participants With Objective Intermediate Sleep Duration

Predictor	Overall Deaths, No. (%)	HR (95% CI)			
		Unadjusted	Age adjusted	Model 1[a]	Model 2[b]
MI as continuous variable	765 (57.0)	0.47 (0.32-0.68)	0.58 (0.40-0.83)	0.59 (0.41-0.86)	0.53 (0.35-0.82)
MI as categorical variable					
Q4 (> 0.08)	157 (53.8)	0.98 (0.81-1.18)	0.95 (0.79-1.15)	0.97 (0.81-1.18)	0.84 (0.69-1.03)
IQR (-0.19-0.08)	384 (53.6)	Reference	Reference	Reference	Reference
Q1 (≤-0.19)	224 (67.1)	1.50 (1.28-1.77)	1.35 (1.15-1.60)	1.34 (1.14-1.59)	1.26 (1.03-1.52)

[a] Model 1 included age, race (white or nonwhite), education, body mass index, smoking status, weekly alcohol consumption, daily caffeine use, antidepressants, benzodiazepines, sleep medications, and site.
[b] Model 2 included Model 1 plus overall arousal index, percentage of sleep time oxygen saturation below 80%, Stage 2%, Stage 3-4%, Stage REM%, Epworth Sleepiness Scale score, Pittsburgh Sleep Quality Index, Teng Mini-Mental State Examination score, Physical Activity Scale for the Elderly score, Geriatric Depression score, congestive heart failure, chronic obstructive pulmonary disease, diabetes, heart attack, stroke, wake after sleep onset, objective total sleep time, hypertension, apnea hypopnea index, periodic limb movements index, habitual bed time, habitual wake-up time, and subjective sleep wellness for the PSG night.
Abbreviation: MI = misperception index.

Table S6 Mortality HRs From Cox Regression by MI for Participants Without Extremely Worse or Better Sleep than Usual

Predictor	Overall Deaths, No. (%)	HR (95% CI) Unadjusted	Age adjusted	Model 1[a]	Model 2[b]
MI as continuous variable	1372 (59.3)	0.47 (0.38-0.58)	0.56 (0.45-0.69)	0.56 (0.45-0.70)	0.65 (0.50-0.85)
MI as categorical variable					
Q4 (> 0.08)	276 (56.3)	1.03 (0.89-1.18)	1.01 (0.88-1.16)	0.97 (0.85-1.12)	0.96 (0.83-1.12)
IQR (-0.19-0.08)	666 (55.3)	Reference	Reference	Reference	Reference
Q1 (≤-0.19)	430 (69.2)	1.52 (1.35-1.71)	1.38 (1.22-1.56)	1.39 (1.23-1.57)	1.28 (1.11-1.47)

[a] Model 1 included age, race (white or non-white), education, body mass index, smoking status, weekly alcohol consumption, daily caffeine use, antidepressants, benzodiazepines, sleep medications, and site.
[b] Model 2 included Model 1 plus overall arousal index, percentage of sleep time oxygen saturation below 80%, Stage 2%, Stage 3-4%, Stage REM%, Epworth Sleepiness Scale score, Pittsburgh Sleep Quality Index, Teng Mini-Mental State Examination score, Physical Activity Scale for the Elderly score, Geriatric Depression score, congestive heart failure, chronic obstructive pulmonary disease, diabetes, heart attack, stroke, wake after sleep onset, objective total sleep time, hypertension, apnea hypopnea index, periodic limb movements index, habitual bed time, habitual wake-up time, and subjective sleep wellness for the PSG night.
Abbreviation: MI = misperception index.

Table S7 Mortality HRs From Cox Regression by MI for Participants Without Subjective Sleep Complaints

Predictor	Overall Deaths, No. (%)	HR (95% CI)			
		Unadjusted	Age adjusted	Model 1[a]	Model 2[b]
MI as continuous variable	863 (56.9)	0.41 (0.31-0.54)	0.51 (0.39-0.66)	0.52 (0.39-0.68)	0.60 (0.42-0.85)
MI as categorical variable					
Q4 (> 0.08)	130 (51.2)	0.94 (0.77-1.14)	0.86 (0.71-1.05)	0.82 (0.67-1.00)	0.86 (0.69-1.06)
IQR (-0.19-0.08)	425 (53.2)	Reference	Reference	Reference	Reference
Q1 (≤-0.19)	308 (66.5)	1.50 (1.30-1.74)	1.36 (1.17-1.57)	1.35 (1.16-1.57)	1.29 (1.08-1.54)

[a] Model 1 included age, race (white or non-white), education, body mass index, smoking status, weekly alcohol consumption, daily caffeine use, antidepressants, benzodiazepines, sleep medications, and site.
[b] Model 2 included Model 1 plus overall arousal index, percentage of sleep time oxygen saturation below 80%, Stage 2%, Stage 3-4%, Stage REM%, Epworth Sleepiness Scale score, Pittsburgh Sleep Quality Index, Teng Mini-Mental State Examination score, Physical Activity Scale for the Elderly score, Geriatric Depression score, congestive heart failure, chronic obstructive pulmonary disease, diabetes, heart attack, stroke, wake after sleep onset, objective total sleep time, hypertension, apnea hypopnea index, periodic limb movements index, habitual bed time, habitual wake-up time, subjective sleep wellness for the PSG night.
Abbreviation: MI = misperception index.

Table S8 Mortality HRs From Cox Regression by MI by Quintiles

Predictor	Overall Deaths, No. (%)	HR (95% CI)			
		Unadjusted	Age adjusted	Model 1[a]	Model 2[b]
MI as categorical variable	1596 (59.7)				
80-100th percentile:>0.12	309 (58.7)	1.15 (0.98-1.35)	1.10 (0.93-1.29)	1.05 (0.89-1.24)	0.97 (0.81-1.15)
60-80th percentile:-0.01-0.12	295 (53.6)	1.01 (0.86-1.19)	0.99 (0.84-1.17)	0.96 (0.81-1.13)	0.93 (0.79-1.10)
40-60th percentile:-0.11--0.01	291 (52.7)	Reference	Reference	Reference	Reference
20-40th percentile:-0.23--0.11	323 (62.5)	1.22 (1.04-1.43)	1.09 (0.93-1.27)	1.05 (0.89-1.23)	1.03 (0.87-1.21)
0-20th percentile: ≤-0.23	378 (71.5)	1.72 (1.47-2.00)	1.44 (1.24-1.68)	1.43 (1.23-1.67)	1.29 (1.09-1.53)

[a] Model 1 included age, race (white or non-white), education, body mass index, smoking status, weekly alcohol consumption, daily caffeine use, antidepressants, benzodiazepines, sleep medications, and site.

[b] Model 2 included Model 1 plus overall arousal index, percentage of sleep time oxygen saturation below 80%, Stage 2%, Stage 3-4%, Stage REM%, Epworth Sleepiness Scale score, Pittsburgh Sleep Quality Index, Teng Mini-Mental State Examination score, Physical Activity Scale for the Elderly score, Geriatric Depression score, congestive heart failure, chronic obstructive pulmonary disease, diabetes, heart attack, stroke, wake after sleep onset, objective total sleep time, hypertension, apnea hypopnea index, periodic limb movements index, habitual bed time, habitual wake-up time, and subjective sleep wellness for the PSG night.

Abbreviation: MI = misperception index.

Table S9 Mortality HRs From Cox Regression by MI After Multiple Imputation

Predictor	Overall Deaths, No. (%)	HR (95% CI)			
		**Unadjusted**	**Age adjusted**	**Model 1[a]**	**Model 2[b]**
MI as continuous variable	1596 (59.7)	0.57 (0.48-0.67)	0.65 (0.55-0.77)	0.66 (0.56-0.78)	0.71 (0.59-0.87)
MI as categorical variable					
Q4 (> 0.08)	374 (57.9)	1.05 (0.93-1.19)	1.04 (0.92-1.17)	1.02 (0.90-1.15)	0.97 (0.85-1.11)
IQR (-0.19-0.08)	750 (55.6)	Reference	Reference	Reference	Reference
Q1 (≤-0.19)	472 (69.6)	1.51 (1.35-1.70)	1.37 (1.22-1.54)	1.38 (1.23-1.56)	1.29 (1.13-1.47)

[a] Model 1 included age, race (white or non-white), education, body mass index, smoking status, weekly alcohol consumption, daily caffeine use, antidepressants, benzodiazepines, sleep medications, and site.

[b] Model 2 included Model 1 plus overall arousal index, percentage of sleep time oxygen saturation below 80%, Stage 2%, Stage 3-4%, Stage REM%, Epworth Sleepiness Scale score, Pittsburgh Sleep Quality Index, Teng Mini-Mental State Examination score, Physical Activity Scale for the Elderly score, Geriatric Depression score, congestive heart failure, chronic obstructive pulmonary disease, diabetes, heart attack, stroke, wake after sleep onset, objective total sleep time, hypertension, apnea hypopnea index, periodic limb movements index, habitual bed time, habitual wake-up time, subjective sleep wellness for the PSG night.

Abbreviation: MI = misperception index.
